# Molecular Mechanisms Underlying Inflammation in Early-Onset Neonatal Sepsis: A Systematic Review of Human Studies

**DOI:** 10.3390/jcm14155315

**Published:** 2025-07-28

**Authors:** Anca Vulcănescu, Mirela-Anișoara Siminel, Anda-Lorena Dijmărescu, Maria-Magdalena Manolea, Sidonia-Maria Săndulescu, Virginia Maria Rădulescu, Valeriu Gheorman, Sorin-Nicolae Dinescu

**Affiliations:** 1 “Filantropia” Clinical Municipal Hospital, 200143 Craiova, Romania; anca.vulcanescu@umfcv.ro (A.V.); magdalena.manolea@umfcv.ro (M.-M.M.); sidonia.sandulescu@umfcv.ro (S.-M.S.); 2 University of Medicine and Pharmacy of Craiova, 200349 Craiova, Romania; 3 Department of Neonatology, University of Medicine and Pharmacy of Craiova, 200349 Craiova, Romania; 4 Department of Obstetrics and Gynecology, University of Medicine and Pharmacy of Craiova, 200349 Craiova, Romania; 5 Department of Medical Informatics and Biostatistics, University of Medicine and Pharmacy of Craiova, 200349 Craiova, Romania; virginia.radulescu@umfcv.ro; 6 Department of Obstetrics and Gynecology, Buna Vestire Clinic, 200345 Craiova, Romania; valeriu_gheorman@yahoo.com; 7 Clinical Emergency County Hospital, 200642 Craiova, Romania; sorin.dinescu@umfcv.ro; 8 Department of Epidemiology, University of Medicine and Pharmacy of Craiova, 200349 Craiova, Romania

**Keywords:** early-onset neonatal sepsis, immune dysregulation, sterile inflammation, biomarkers, neonatal immune response

## Abstract

**Background/Objective**: Early-onset neonatal sepsis (EOS), defined as infection occurring within the first 72 h after birth, remains a major contributor to neonatal morbidity and mortality worldwide. Although advances in perinatal care have improved overall outcomes, the diagnosis of EOS continues to be challenging. Clinical presentations are often nonspecific, laboratory confirmation is often delayed, and immune responses vary considerably among neonates. Expanding our understanding of the molecular mechanisms underlying EOS is essential in enhancing early detection, refining risk stratification, and guiding therapeutic strategies. This systematic review aims to synthesize the available information on the molecular pathways involved in EOS, focusing on pathogen-induced inflammation, systemic immune responses, sterile inflammatory processes, interactions between infectious and non-infectious pathways, as well as emerging molecular diagnostic approaches. **Methods**: A comprehensive review of original research articles and reviews published between January 2015 and January 2025 was conducted; studies were included based on their focus on human neonates and their analysis of molecular or immunological mechanisms relevant to EOS pathogenesis, immune dysregulation, or novel diagnostic strategies. **Results**: Pathogen-driven inflammation typically involves the activation of Toll-like receptors (TLRs), the recruitment of neutrophils, and the release of pro-inflammatory cytokines such as IL-6, IL-1β, and TNF-α, particularly in response to vertical transmission of organisms like Escherichia coli and Streptococcus agalactiae. Systemic inflammatory responses are marked by cytokine dysregulation, contributing to multi-organ dysfunction. Sterile inflammation, often initiated by hypoxia–reperfusion injury or intrauterine stress, amplifies susceptibility to sepsis. Interactions between immune, metabolic, and endothelial pathways further exacerbate tissue injury. Recent advances, including transcriptomic profiling, microRNA-based biomarkers, and immune checkpoint studies, offer promising strategies for earlier diagnosis and individualized therapeutic options. **Conclusions**: EOS arises from a complex interplay of infectious and sterile inflammatory mechanisms. A deeper molecular understanding holds promise for advancing correct diagnostics and targeted therapies, aiming to improve neonatal outcomes.

## 1. Introduction

Early-onset neonatal sepsis (EOS), defined as sepsis occurring within the first 72 h of life, remains a leading cause of mortality and long-term morbidity among neonates, particularly those who were born preterm or with low birth weight [1,2]. Despite significant advances in perinatal and neonatal intensive care, EOS continues to present challenges in both diagnosis and management, due to clinical signs being often subtle and nonspecific, laboratory confirmation typically being delayed, and disease progression being rapid and unpredictable [3,4].

EOS commonly arises from vertical transmission of bacterial pathogens during labor or shortly before birth. Group B Streptococcus (GBS) and Escherichia coli remain the most frequently implicated organisms, known for expressing surface virulence factors that facilitate adhesion to mucosal surfaces, tissue invasion, and immune evasion [5,6]. In response to these pathogens, neonatal innate immune pathways are activated primarily through pattern recognition receptors (PRRs), with Toll-like receptors (TLRs) playing a central role. This leads to the production of pro-inflammatory cytokines, including interleukin-6 (IL-6), interleukin-1β (IL-1β), and tumor necrosis factor-alpha (TNF-α) [7,8,9]. However, the neonatal immune system is inherently immature; therefore, reduced neutrophil activity, lower complement concentrations, and a predisposition toward immune tolerance weaken the host’s defense against infection [10,11].

Recent studies have emphasized the role of sterile inflammation—immune activation without direct infection—in the pathogenesis of EOS [12,13,14]. Insults such as hypoxia–reperfusion injury, mechanical ventilation, and antenatal inflammation can lead to the release of damage-associated molecular patterns (DAMPs), including high-mobility group box 1 (HMGB1), adenosine triphosphate (ATP), and mitochondrial DNA. These molecules can amplify inflammatory responses in the absence of pathogens, complicating the diagnostic picture [15,16,17].

Moreover, systemic inflammation in EOS reflects broader dysregulation across multiple systems. Apart from cytokine imbalance, complex interactions between the immune, metabolic, and endothelial pathways have been documented [18,19]. Endothelial injury, coagulation disturbances, and mitochondrial dysfunction are increasingly recognized as key elements of disease progression and multisystem organ failure in neonatal sepsis [20,21,22]. This complex pathophysiology blurs the distinction between infectious and non-infectious etiologies, making clinical decision-making even more challenging [23].

Advances in molecular diagnostics are beginning to offer new possibilities for improving the early detection of EOS. Techniques such as transcriptomic profiling, microRNA analysis, proteomics, and immune checkpoint profiling have revealed novel biomarkers and potential therapeutic targets [24,25]. Recent research has identified distinct sepsis-associated gene signatures, dysregulated microRNAs, and emerging cell surface markers that could enhance risk stratification and contribute to the development of individualized treatment strategies.

Therefore, this systematic review aims to answer the following research question: What are the key molecular mechanisms involved in the pathogenesis of early-onset neonatal sepsis (EOS), as reported in human studies published over the last decade?

Our hypothesis is that early-onset neonatal sepsis arises from a complex interplay between infectious and non-infectious (sterile) inflammatory triggers, reflected at the molecular level through dysregulated immune, endothelial, and metabolic pathways.

The objective of this review is to synthesize the current evidence on the molecular and immunological mechanisms involved in EOS, including pathogen-induced pathways, sterile inflammation, systemic immune responses, and emerging diagnostic strategies. By doing so, we aim to highlight potential molecular biomarkers and targets for precision-based neonatal sepsis management.

## 2. Materials and Methods

### 2.1. Study Design

**Figure 1:**
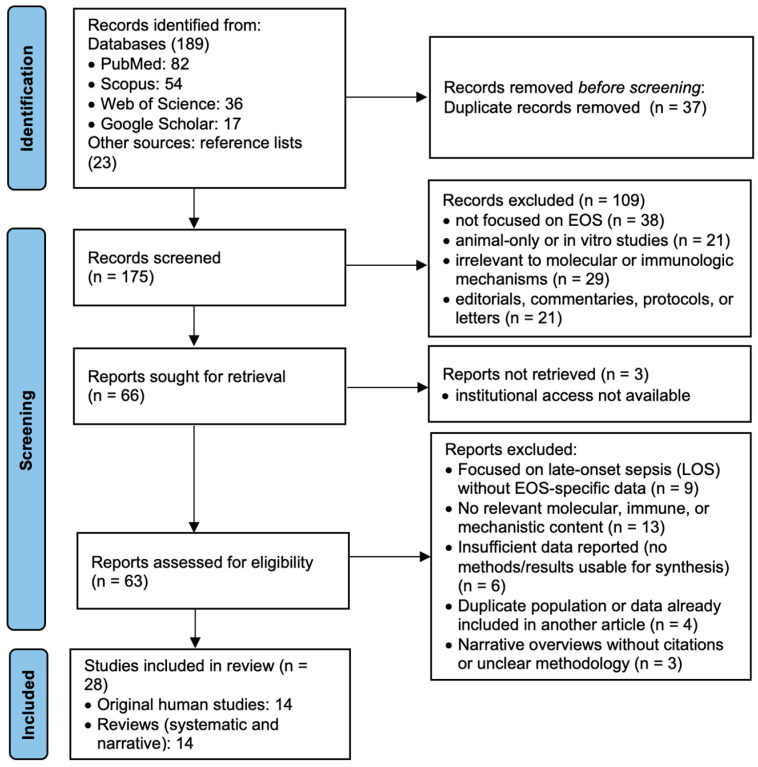
Prisma flowchart.

This systematic review was conducted following the Preferred Reporting Items for Systematic Reviews and Meta-Analyses (PRISMA) 2020 guidelines (Supplementary Materials S3). A comprehensive search of the literature was carried out across PubMed, Scopus, and Web of Science databases, including studies published from January 2015 to January 2025. Additional sources included Google Scholar searches and manual screening of reference lists from relevant papers, as illustrated in Figure 1. The final search in each database was performed on 31 January 2025, using the same keyword strategy across PubMed, Scopus, and Web of Science. Manual searches and screening of reference lists were also finalized on this date.

Two reviewers independently screened the titles and abstracts for relevance, and full texts were retrieved for articles meeting the initial criteria. Any disagreements regarding study inclusion were resolved through discussion. The titles and abstracts were independently assessed by two reviewers based on the predefined eligibility criteria. Full-text articles were, likewise, independently evaluated by the same two reviewers. In case of disagreement, a third reviewer was consulted to reach consensus. Data extraction was performed manually and cross-verified by the reviewers for accuracy and consistency.

The search strategy incorporated both Medical Subject Headings (MeSH) and free-text keywords, including “neonatal sepsis”, “early-onset neonatal sepsis”, and “EOS”, combined with terms such as “molecular mechanism”, “immune response”, “biomarkers”, “Toll-like receptors”, “cytokines”, “gene expression”, and “sterile inflammation”.

For example, the full PubMed search query used was as follows: (“neonatal sepsis” [MeSH Terms] OR “early-onset sepsis” OR “EOS”) AND (“molecular mechanism” OR “immune response” OR “inflammation” OR “cytokines” OR “biomarkers” OR “gene expression” OR “Toll-like receptor” OR “transcriptome”). A detailed description of all search strings and database strategies is provided in the Supplementary Materials S1.

### 2.2. Eligibility Criteria

The inclusion criteria used were the following:

- Population: human studies involving neonates (≤72 h old) with either confirmed or suspected early-onset sepsis.
- Content: articles that described molecular mechanisms, biomarkers, immune signaling pathways, or host–pathogen interaction related to EOS.
- Study type: original research articles and review articles.
- Period: published between 1 January 2015 and 31 January 2025.
- Language: English.
- Setting: studies conducted in hospitals or clinical laboratories, or translational research settings involving human neonatal samples (e.g., cord blood, serum, cells).

The exclusion criteria used were the following:

- Studies focused exclusively on late-onset sepsis (LOS).
- Studies that were based solely on animal models or in vitro experiments without direct human relevance.
- Reported clinical outcomes without addressing molecular, immunological, or mechanistic data.
- Studies which were categorized as editorials, letters, or commentaries without original data or formal review.

Risk of bias was evaluated manually, based on study design, sample size, clarity of outcome reporting, disclosure of funding sources, and declaration of potential conflicts of interest. Each study presented either a low (15 studies) or moderate risk of bias (13 studies), mainly due to incomplete outcome reporting or missing disclosures regarding funding and conflicts of interest. The risk of bias was assessed qualitatively using five criteria: (1) study design rigor (e.g., prospective vs. retrospective); (2) sample size adequacy (relative to objectives); (3) clarity and transparency in reporting outcomes; (4) disclosure of funding sources; and (5) declaration of potential conflicts of interest. Studies that met at least four of the five criteria were classified as “low risk”, while those meeting only two or three criteria were classified as “moderate risk”. No study was excluded based on high risk, as no included article met fewer than two criteria. Formal assessment of publication bias was not conducted, as the heterogeneity of study designs and the narrative nature of this review precluded the use of quantitative methods typically employed for such evaluation.

Most original research articles and review papers demonstrated adequate methodological transparency and maintained strong relevance to the review’s objectives, supporting their inclusion in the final synthesis. A detailed summary of the risk of bias assessments is presented in both tabular and narrative formats (see the Supplementary Materials S2).

A qualitative sensitivity analysis showed that exclusion of moderate-risk studies did not materially affect the main findings, confirming the robustness of the results. Specificity was maintained by limiting inclusion to studies involving human neonates with clearly defined early-onset sepsis (≤72 h of life). Although sample sizes, outcome measures, and analytical techniques varied across studies, the thematic consistency observed supported the use of narrative synthesis. Due to variability in study designs, formal statistical measures of heterogeneity were not calculated.

Data extraction focused on several key elements, including study type (original research or review), year and country of publication, population characteristics, specific molecular targets or pathways investigated, EOS-specific relevance, and key findings (Table 1). Each eligible article was subsequently categorized into one or more thematic domains: pathogen-induced inflammation, systemic inflammation, sterile inflammatory responses, pathway interactions, and emerging molecular perspectives (Table 2).

For classification purposes, the EOS-specific relevance of each study was determined using four categories:

(1) Direct—studies that explicitly addressed molecular or immunological mechanisms in early-onset neonatal sepsis (≤72 h).
(2) Partial—studies that included both EOS and other forms of neonatal sepsis but presented stratified or relevant data for EOS.
(3) Indirect—studies addressing related molecular pathways in neonatal immune responses without a clear focus on EOS.
(4) Conceptual—theoretical or narrative articles discussing inflammation or sepsis frameworks applicable to the EOS context.

These categories were applied during full-text evaluation and were not defined a priori.

Thematic categorization of studies into five mechanistic domains was performed post hoc, based on full-text review. Each study was assigned to one or more categories according to its molecular focus, regardless of the study type (original or review).

To maintain alignment with the review’s objectives, studies that did not specifically address molecular mechanisms relevant to early-onset sepsis were excluded. For example, the study by Bethou and Bhat (2022) [26] provided a valuable clinical view of neonatal sepsis within an Indian tertiary care setting, but lacked focus on molecular pathways and early-onset sepsis. Similarly, Chauhan et al. (2017) [27] presented a broad review of potential biomarkers for neonatal infections, but did not differentiate early-onset cases and included data from non-human models, making their review unfit for inclusion. These exclusions ensured that the final set of included studies remained tightly focused on the chosen topic.

For data organization and simple descriptive statistics, Microsoft Excel (Version 16.93.1) and macOS Numbers (Version 14.3) were utilized. No specialized software for bias assessment were employed.

**Table 1:** Review findings.

Author(s)	Study Type	Year and Country	Population Characteristics	Sample Size	EOS Definition	Molecular Targets/Pathways	EOS-Specific Relevance	Key Findings
Marchant et al. [28]	Original	2015, Canada	Preterm neonates	45 p	Clinical EOS, based on CDC guidelines	TLR2, TLR4, cytokines (IL-6, TNF-α)	Direct	Preterm neonates show impaired TLR responses and cytokine production.
Nakstad et al. [29]	Original	2016, Norway	Cord blood model	30	In vitro stimulation model mimicking EOS	TLRs, IL-6	Direct	Cord blood IL-6 response enables early GBS detection.
Dias et al. [30]	Review	2021, Ireland	Narrative—humans	Not applicable	Not uniformly specified across studies included	TLR signaling	Direct	TLR pathways are promising targets for neonatal immune modulation.
Shane et al. [31]	Review	2017, USA	Neonates—global data	N/A	Mixed (includes EOS and LOS, not always specified)	Immune mediators	Direct	Summarizes immune deficits, diagnostics, and pathogens in EOS.
Dong & Speer [32]	Review	2015, Germany	Neonatal sepsis, all types	N/A	Includes both EOS and LOS, focus more on LOS	Immune maturation	Partial	Describes immune development affecting sepsis response.
Sweeney et al. [33]	Review	2017, Australia	Perinatal infections	N/A	Broad perinatal infection model; no strict EOS separation	Microbial virulence factors	Partial	Details pathogen virulence mechanisms relevant to perinatal sepsis.
Raymond et al. [34]	Original	2017, USA	Neonates, immune suppression	36	Clinical EOS (culture not required)	Cytokines, immune exhaustion	Direct	Highlights immune exhaustion and cytokine dysregulation in EOS.
Khaertynov et al. [35]	Original	2017, Russia	Neonates with EOS vs. LOS	58 (30 EOS, 28 LOS)	Culture-confirmed EOS (within 72 h)	Cytokines (IL-6, TNF-α)	Direct	EOS features elevated IL-6 and TNF-α vs. LOS.
Wynn & Wong [36]	Review	2016, USA	Theoretical/Review	N/A	Not applied to specific patients	TLRs, NF-κB	Indirect	Explains role of TLRs and NF-κB in inflammation.
Ershad et al. [37]	Review	2019, USA	General neonatal population	N/A	Includes sepsis in neonates; not limited to EOS	General cytokines	Partial	Describes cytokine profiles in neonatal infections.
Dong [38]	Review	2019, Germany	Narrative neonatal focus	N/A	Includes EOS and general neonatal sepsis	Inflammation and immunity	Partial	Reviews inflammation and immune mechanisms in neonatal sepsis.
Hibbert et al. [39]	Original	2018, Australia	Preterm and term neonates	42	Clinical diagnosis, based on signs and markers	Sepsis-induced immunosuppression	Direct	Sepsis induces immune suppression even in early phases.
Moon et al. [40]	Original	2021, South Korea	FGR and chorioamnionitis neonates	40	Clinical EOS based on signs and inflammatory markers	DAMPs, sterile inflammation	Direct	FGR and inflammation predispose neonates to EOS.
Vincent [41]	Review	2023, Belgium	Theoretical/discussion	N/A	Not neonatal-specific; discusses sepsis broadly	Sepsis/infection distinction	Conceptual	Distinguishes systemic inflammation from infection in sepsis.
Wynn & Polin [42]	Review	2018, USA	Neonates with suspected EOS	N/A	Includes suspected EOS; focus on definitions and frameworks	Consensus definitions	Direct	Highlights needed for EOS-specific criteria in research.
Conti et al. [43]	Review	2020, Italy	Human neonatal immune pathways	N/A	Focused on neonatal inflammation; EOS included as context	Immunometabolism, TLRs	Direct	Explores metabolic modulation of inflammation in EOS.
Parra-Llorca et al. [44]	Original	2023, Spain	Preterm neonates with EOS	52	Confirmed or suspected EOS based on clinical and microbiological data	Immune response, ROS	Direct	EOS alters microbiome, immune response, and ROS signaling.
Tsantes et al. [45]	Review	2023, Greece	Neonates with coagulopathy	N/A	Includes EOS as one of several contexts for coagulation	Coagulation pathways	Direct	Reviews EOS-related coagulopathy mechanisms.
Gialamprinou et al. [46]	Original	2023, Greece	EOS patients, NICU	28 EOS cases vs. 20 controls	Culture-confirmed EOS	Coagulation, Gram-positive EOS	Direct	Finds Gram-positive EOS associated with platelet dysfunction.
Hensler et al. [47]	Original	2022, USA	Neonatal immune checkpoint focus	34	Clinical and laboratory-confirmed EOS	Immune checkpoints	Direct	Identifies immune checkpoints as regulators in EOS.
Yan & Zhou [48]	Original	2022, China	Sepsis dataset—clinical validation	56 (bioinformatics set) + 24 validation cases	Included EOS-specific analysis based on timing and markers	mRNA biomarkers	Direct	Integrates bioinformatics with clinical data to identify key markers.
Luo et al. [49]	Original	2023, China	Transcriptome in EOS neonates	60	Confirmed EOS by clinical/lab criteria	DEGs, immune infiltration	Direct	Identifies DEGs linked to inflammation in EOS.
Celik et al. [50]	Review	2022, Turkey	Narrative EOS review	N/A	Comprehensive review focused on EOS	General inflammatory pathways	Direct	Summarizes EOS immune mechanisms and diagnostics.
Ruan et al. [51]	Review	2018, China	Systematic review, neonates	Meta-analysis (15 studies, >1000 neonates)	Defined EOS as sepsis within 72 h of life	Presepsin, CRP	Direct	Supports presepsin and CRP as EOS biomarkers.
Jouza et al. [52]	Original	2022, Czech Republic	Neonatal blood samples	46 neonates (23 EOS, 23 controls)	Culture-confirmed and clinical EOS	miRNA biomarkers	Direct	miRNAs hold promise as EOS biomarkers.
Ng et al. [53]	Review	2015, Hong Kong	Review of lab biomarkers	N/A	Includes EOS cases; not always separated from LOS	CRP, IL-6, PCT	Direct	Evaluates CRP, IL-6, and PCT in EOS monitoring.
Chauhan et al. [27]	Review	2017, India	Narrative review EOS biomarkers	N/A	Not strictly separated; includes experimental and clinical data	Immune and lab markers	Direct	Reviews emerging EOS biomarkers in clinical settings.
Pietrasanta et al. [54]	Review	2019, Italy	Review of vascular function in EOS	N/A	Includes EOS in the context of endothelial injury	Endothelial dysfunction	Direct	Links endothelial injury to EOS pathogenesis.

**Table 2:** Review findings—domain division.

Pathogen-Induced Inflammation	Systemic Inflammation	Sterile Inflammation	Interactions Between Mechanisms	Emerging Molecular Perspectives
Marchant et al., 2015 [28]	Raymond et al., 2017 [34]	Moon et al., 2021 [40]	Wynn & Polin, 2018 [42]	Yan & Zhou, 2022 [48]
Nakstad et al., 2016 [29]	Khaertynov et al., 2017 [35]	Vincent, 2023 [41]	Conti et al., 2020 [43]	Luo et al., 2023 [49]
Dias et al., 2021 [30]	Wynn & Wong, 2016 [36]	Hensler et al., 2022 [47]	Celik et al., 2022 [50]	Ruan et al., 2018 [51]
Shane et al., 2017 [31]	Ershad et al., 2019 [37]		Pietrasanta et al., 2019 [54]	Jouza et al., 2022 [52]
Dong & Speer, 2015 [32]	Dong, 2019 [38]			Ng et al., 2015 [53]
Sweeney et al., 2017 [33]	Hibbert et al., 2018 [39]			Chauhan et al., 2017 [27]
	Parra-Llorca et al., 2023 [44]			
	Tsantes et al., 2023 [45]			
	Gialamprinou et al., 2023 [46]			

## 3. Results

The final set of 28 studies was organized according to study characteristics and relevance to early-onset neonatal sepsis (EOS). Table 1 provides a structured summary of each study’s design, population characteristics, sample size (if applicable), EOS definition, molecular pathways investigated, EOS-specific relevance, and key findings.

Studies were classified according to their EOS-specific relevance—direct, partial, indirect, or conceptual—as detailed in the Materials and Methods section. Diagnostic definitions varied, ranging from strict culture-confirmed EOS to broader clinical criteria or experimental models. Original studies often investigated immune signaling, transcriptomic profiles, or biomarker performance, while reviews offered integrative frameworks or conceptual models.

The table highlights key molecular targets involved in pathogen recognition, cytokine cascades, endothelial dysfunction, immunometabolic regulation, and novel biomarker discovery (e.g., miRNAs, checkpoint proteins). This comparative synthesis emphasizes both the diversity of research approaches and the molecular complexity of EOS.

To support thematic synthesis, Table 2 groups these studies into five mechanistic domains: pathogen-induced inflammation, systemic inflammation, sterile inflammation, pathway interactions, and emerging molecular perspectives.

Table 2 presents the thematic categorization of the included studies, based on their primary molecular focus. This post hoc classification was developed through full-text review and includes five mechanistic domains: pathogen-induced inflammation, systemic inflammation, sterile inflammation, pathway interactions, and emerging molecular perspectives.

Each study was assigned to one or more domains depending on the molecular mechanisms or signaling pathways investigated, irrespective of study design (original research or review). This classification aimed to support thematic synthesis across diverse study types. Reference numbers are provided to ensure transparency and facilitate traceability.

This classification shows that most studies converged on pathogen recognition and systemic inflammatory responses, particularly involving cytokine cascades, innate immune receptors, and classical biomarkers. However, a growing number of studies explored sterile inflammation, immunometabolic dysregulation, and vascular involvement in EOS pathophysiology. The presence of studies across multiple categories reflects the interconnected nature of inflammatory mechanisms in neonates and underscores the value of integrative approaches in EOS research.

### 3.1. Pathogen-Induced Inflammation

Pathogen-induced inflammation remains a central mechanism in the onset of early-onset neonatal sepsis (EOS), initiating immune activation through the recognition of microbial structures by pattern recognition receptors (PRRs). Among the 28 studies included in this review, 12 directly explored this pathway, underscoring the pivotal role of Toll-like receptor (TLR)-mediated signaling and bacterial virulence factors in triggering neonatal immune responses.

#### 3.1.1. Toll-like Receptor Signaling in Neonates

Several studies identified TLRs, particularly TLR2 and TLR4, as critical sensors for pathogen-associated molecular patterns (PAMPs) in the neonatal immune system. One study demonstrated that preterm neonates exhibit reduced expression of TLRs and impaired downstream signaling, resulting in diminished activation of NF-κB and lower levels of pro-inflammatory cytokines such as interleukin-6 (IL-6) and tumor necrosis factor-alpha (TNF-α). Other authors, using a cord blood stimulation model, observed delayed IL-6 production following the exposure to Streptococcus agalactiae, highlighting the functional immaturity of neonatal innate defenses [28,29].

#### 3.1.2. Bacterial Virulence and Barrier Invasion

In addition, immune immaturity and microbial virulence mechanisms were identified as major contributors to EOS pathogenesis. It is described how Group B Streptococcus and Escherichia coli utilize adhesins, internalins, and hyaluronidase enzymes to breach mucosal and epithelial barriers, facilitating systemic invasion. Starting from this hypothesis, other authors explored the role of Ureaplasma species in inducing low-grade, chronic inflammation that may predispose neonates to sepsis. These bacterial strategies are often complemented by immune evasion tactics, including capsule-mediated resistance to phagocytosis and molecular mimicry [32,33].

#### 3.1.3. Cytokine Response and Immune Amplification

Following TLR engagement, downstream signaling activates the release of pro-inflammatory mediators. Some authors reported that elevated levels of IL-1β, IL-6, and TNF-α not only function as immune effectors but also contribute to endothelial dysfunction, increased vascular permeability, and metabolic disturbances when produced excessively. Another study identified a two-phase immune response, where an initial hyperinflammatory phase can transition to subsequent immunosuppression, intensifying the risk of secondary infections and organ dysfunction [31,34,38].

#### 3.1.4. Diagnostic Implications and Targeted Modulation

Emerging evidence suggests that TLRs and their downstream signaling pathways could serve as both diagnostic biomarkers and therapeutic targets in EOS. Some suggested that differential immune activation patterns between Gram-positive and Gram-negative pathogens may enable the development of pathogen-specific molecular diagnostics; such advances could facilitate earlier detection and allow for more targeted therapeutic strategies, moving beyond empirical antibiotic administration [30,43].

In recent years, several promising diagnostic tools have been investigated in neonatal populations. For instance, presepsin (sCD14-ST) has shown potential for EOS detection with higher specificity than CRP or IL-6, and is now being evaluated in multicenter trials (Klingenberg et al. [55]). Additional biomarkers such as pentraxin 3 (PTX3) and CD64 expression on neutrophils are also emerging as rapid-response indicators of neonatal infection severity (Ng et al. [56]; El-Badawy et al. [57]). On the therapeutic front, experimental approaches involving TLR4 antagonists, IL-1 and IL-6 blockade, and modulation of PD-1/PD-L1 signaling are under preclinical investigation for potential use in neonatal inflammatory control. These developments mark a shift toward precision-based sepsis management in neonatology, where molecular profiles may soon guide both diagnosis and targeted immunomodulation.

### 3.2. Systemic Inflammation

Systemic inflammation represents a main consequence following pathogen recognition and immune activation in EOS. It is characterized by a cascade involving cytokine release, endothelial dysfunction, coagulation abnormalities, and progressive multi-organ injury. Across the 28 studies reviewed, 15 specifically investigated systemic immune responses and their physiological consequences, identifying immunological patterns and highlighting mediators critical to disease progression.

#### 3.2.1. Cytokine Storm and Immune Dysregulation

Elevated concentrations of pro-inflammatory cytokines, including interleukin-6 (IL-6), interleukin-1β (IL-1β), tumor necrosis factor-alpha (TNF-α), and procalcitonin, were reported as symbols of systemic inflammation in EOS. One study found that IL-6 and TNF-α levels were significantly higher in EOS compared to late-onset sepsis, particularly within the first 48 h of life, reinforcing their value for early diagnosis. Similarly, other authors emphasized the importance of dynamic cytokine monitoring, suggesting that tracking these biomarkers over time can improve diagnostic specificity and potentially predict disease severity [35,44,52].

Recent studies have expanded our understanding of the neonatal cytokine response beyond classical pro-inflammatory mediators. For instance, emerging evidence suggests that the dysregulation of IL-18 and the NLRP3 inflammasome complex may also play a critical role in EOS pathogenesis, particularly in preterm neonates with heightened innate responses (El-Badawy et al. [57]). Moreover, anti-inflammatory mediators such as IL-10 appear to be insufficiently expressed in septic neonates, potentially contributing to sustained systemic inflammation and poor resolution (Wang et al. [58]). These insights align with growing interest in immune checkpoint regulators like PD-1 and CTLA-4, which may be overexpressed in neonatal sepsis and linked to immune paralysis (Dong et al. [32]; Wang et al. [59]).

While cytokine profiling has not yet entered routine neonatal diagnostics, recent advances in multiplex assays and microfluidic platforms hold promise for early risk stratification and personalized therapeutic targeting.

#### 3.2.2. Endothelial Injury and Microcirculatory Instability

Recent data suggest that endothelial glycocalyx degradation plays a central role in the pathogenesis of neonatal sepsis. Biomarkers such as syndecan-1 and angiopoietin-2 are currently being evaluated for their utility in monitoring endothelial injury and predicting clinical outcomes (Ng et al. [56]; Zonneveld et al. [60]). In parallel, preclinical studies targeting vascular stabilization—using agents such as angiopoietin analogs or sphingosine-1-phosphate modulators—have shown potential to preserve microvascular integrity. These strategies are of particular relevance in preterm infants, where endothelial immaturity may intensify systemic inflammation and microcirculatory dysfunction.

#### 3.2.3. Epigenetic and Transcriptomic Regulation

Recent transcriptomic studies have identified gene expression profiles associated with poor outcomes in neonates with sepsis, including upregulation of IL-1 signaling, neutrophil chemotaxis, and apoptosis-related pathways (Xing et al. [61]; Wang et al. [59]). Moreover, microRNAs such as miR-150 and miR-223 have been implicated as both diagnostic biomarkers and regulators of innate immune activation in early-onset sepsis (Ng et al. [56]; El-Badawy et al. [57]). These findings support the potential use of transcriptomic signatures in guiding risk stratification and individualized interventions. Although still experimental, transcriptome-informed decision making may shape the future of neonatal sepsis care by enabling earlier and more precise immune modulation.

#### 3.2.4. Sepsis-Induced Immunosuppression

Beyond the initial hyperinflammatory phase, several studies reported elements consistent with immune suppression even during early stages of EOS. A decrease in monocyte HLA-DR expression was described, as well as an impaired neutrophil function, and diminished T-cell responses. These findings support the concept of a two-phase immune path, where an early overwhelming inflammatory response is followed by compensatory immunosuppression, increasing vulnerability to secondary infections and delaying recovery [34,39].

#### 3.2.5. Endothelial Dysfunction and Coagulopathy

The systemic inflammatory response in EOS also had important effects on vascular integrity and coagulation systems. Two studies documented evidence of endothelial activation, thrombocytopenia, and platelet consumption in affected neonates. The inflammatory setting promotes the development of disseminated intravascular coagulation (DIC), impairing tissue perfusion and contributing to the evolution of multi-organ dysfunction, a trait of severe neonatal sepsis [45,46].

#### 3.2.6. Organ-Specific Sequelae

The effects of systemic inflammation extend across multiple organ systems, with reviews highlighting pulmonary complications such as acute respiratory distress syndrome (ARDS), alongside renal hypoperfusion and cerebral hypoxia, as frequent sequelae of EOS-associated inflammation. The interaction between vasodilation, capillary leak, and metabolic dysregulation increases tissue injury, exacerbating clinical decline, particularly among preterm and critically ill neonates [36,37].

### 3.3. Sterile Inflammation

Sterile inflammation is increasingly recognized as a significant contributor to the pathogenesis of EOS, particularly in cases where microbiological confirmation of the infection is absent. This immune activation, occurring independently of pathogens, is determined by tissue injury, hypoxic stress, and the release of damage-associated molecular patterns (DAMPs). Seven studies examined the molecular involvement of sterile inflammation in EOS, emphasizing both its diagnostic challenges and clinical significance.

#### 3.3.1. Triggers and Cellular Injury

Sterile inflammatory responses in neonates are often initiated by perinatal physiological insults, including fetal growth restriction (FGR), intrauterine hypoxia, asphyxia, and interventions such as mechanical ventilation. One author demonstrated that neonates born with FGR and histologic evidence of chorioamnionitis exhibit exaggerated postnatal inflammatory responses, despite negative culture findings. These observations suggest that antenatal stressors may lead the neonatal immune system to a hyperinflammatory state, independent of microbial exposure [40].

#### 3.3.2. Role of DAMPs and Inflammasome Activation

Essential to the propagation of sterile inflammation are DAMPs, including high-mobility group box 1 (HMGB1), mitochondrial DNA (mtDNA), extracellular ATP, and S100 proteins. These molecules interact with receptors such as Toll-like receptor 9 (TLR9), the NOD-like receptor protein 3 (NLRP3) inflammasome, and the receptor for advanced glycation end products (RAGE), amplifying cytokine release. Although not all studies directly measured DAMP levels, discussions highlighted the role of inflammasome activation in neonatal immune dysregulation, suggesting that aberrant NLRP3 signaling may guide uncontrolled inflammation and cell injury even in the absence of infection [41,47].

#### 3.3.3. Immune Checkpoints and Regulatory Failure

The regulatory role of immune checkpoint proteins in sterile inflammation was explored, focusing on molecules such as PD-1, CTLA-4, and TIM-3. Typically involved in lightening immune activation, these checkpoints appeared dysfunctional or suppressed in neonates experiencing sterile inflammatory states. Loss of checkpoint regulation may allow persistent cytokine production and sustained tissue damage, providing a mechanical explanation for prolonged inflammation in pathogen-negative EOS cases [47].

#### 3.3.4. Diagnostic and Therapeutic Implications

Differentiating sterile from pathogen-driven inflammation remains a clinical challenge. The urgent need for diagnostic tools capable of distinguishing infectious from non-infectious inflammatory triggers was emphasized, in order to limit unnecessary antibiotic exposure. While traditional biomarkers such as C-reactive protein (CRP) and procalcitonin (PCT) are elevated in both contexts, emerging molecular profiles—particularly DAMP-associated gene expression patterns and inflammasome-related signatures—may enhance diagnostic specificity in future EOS management strategies [41].

### 3.4. Interactions Between Infectious and Sterile Inflammatory Pathways

Emerging evidence suggests that EOS does not result only from microbial invasion or sterile inflammation, rather arising from a complex and often synergistic interaction between pathogen-associated and damage-associated inflammatory mechanisms. Six studies examined how infection-related (PAMP) and non-infectious (DAMP) pathways interact, amplifying one another, and influencing the clinical course of EOS.

#### 3.4.1. Dual Activation of Inflammatory Pathways

Pathogen exposure initiates innate immune activation primarily through recognition of PAMPs by Toll-like receptors (TLRs), as established across multiple studies. However, two authors highlighted that concomitant tissue injury stemming from hypoxia, ischemia–reperfusion events, or birth trauma can lead to the release of endogenous DAMPs, including mitochondrial DNA, ATP, and HMGB1. These molecules engage additional immune receptors such as TLR9, RAGE, and the NLRP3 inflammasome, augmenting and sustaining the inflammatory response initiated by microbial signals [38,42].

#### 3.4.2. Synergistic Cytokine Amplification

The combined activation of PAMP and DAMP pathways results in enhanced cytokine production. Several studies reported that elevated levels of IL-6, TNF-α, and IL-1β are observed not only in response to microbial infection but also in neonates exposed to antenatal or perinatal stressors. An immunometabolic model was proposed, suggesting that inflammation driven simultaneously by PAMPs and DAMPs promotes energy depletion, oxidative stress, and organ dysfunction, establishing a self-perpetuating inflammatory cycle [43].

#### 3.4.3. Implications for Barrier Integrity and Immune Priming

The interaction of infectious and sterile inflammatory signaling also appears to alter epithelial and endothelial barrier functions. Reviews described degradation of the endothelial glycocalyx and increased vascular permeability as effects of combined PAMP–DAMP activation. Such disruptions facilitate microbial translocation and systemic dissemination, explaining why neonates exposed to fetal distress or preterm birth conditions may develop EOS rapidly, even with relatively modest levels of microbial colonization. Moreover, DAMP-mediated immune priming may sensitize innate immune cells, amplifying their reactivity to subsequent microbial challenges [50,54].

#### 3.4.4. Diagnostic Complexity and Clinical Overlap

Clinically, the interplay between sterile and pathogen-induced inflammation complicates both diagnosis and treatment. As some studies have noted, conventional biomarkers such as C-reactive protein (CRP) and procalcitonin lack the specificity to distinguish infectious from non-infectious inflammation, particularly during the early stages of EOS. This diagnostic overlap stresses the need for next-generation biomarkers that can more accurately reflect the dual nature of the inflammatory response in affected neonates [41,53].

### 3.5. Emerging Molecular Perspectives

Recent advances in transcriptomics, biomarker discovery, and systems biology have opened new paths for the early detection and mechanical understanding of EOS. Nine studies included examined emerging molecular approaches with potential diagnostic, prognostic, and therapeutic applications, reflecting a wider shift from conventional clinical markers toward precision immunology in neonatal care.

#### 3.5.1. Transcriptomic and Bioinformatic Profiling

Two original studies utilized transcriptomic analyses and immune cell infiltration profiling to characterize EOS at the molecular level. Their bioinformatic assessments of neonatal datasets identified differentially expressed genes (DEGs) associated with neutrophil activation, chemokine signaling, oxidative phosphorylation, and TLR pathway engagement. Both groups validated their findings against clinical samples, providing early evidence that transcriptomic signatures may differentiate EOS from non-infectious inflammatory conditions. These studies highlight the possibility of integrating multi-omics approaches into research, offering mechanical insights into immune dysregulation and facilitating the stratification of sepsis subtypes based on immune phenotypes [48,49].

#### 3.5.2. Diagnostic Biomarkers: CRP, Presepsin, and miRNAs

Several studies reexamined the utility of established and emerging biomarkers in the diagnosis of EOS. A meta-analysis was conducted, confirming that combining presepsin and C-reactive protein (CRP) measurements improves diagnostic accuracy, particularly in early stages. One study strengthened the use of serial CRP and procalcitonin (PCT) measurements to enhance diagnostic sensitivity. In addition, dysregulated circulating microRNAs (miRNAs) associated with immune regulation and cytokine signaling in neonates with EOS were identified, suggesting their potential as early, minimally invasive biomarkers to complement traditional tests, especially in high-risk populations [51,52,53].

#### 3.5.3. Immune Checkpoints and Systems-Level Modulation

An emerging field involves the role of immune checkpoint proteins, including PD-1 and CTLA-4, in modulating neonatal inflammatory responses. Their findings suggest that therapeutic modulation of checkpoint activity could offer a strategy to soften excessive cytokine release without compromising essential host defenses. While this approach has been widely applied in oncology, its translation to neonatal immunology offers a novel perspective for EOS [47].

#### 3.5.4. Toward Personalized Neonatal Sepsis Care

Building on these molecular insights, reviews emphasized the need to integrate molecular tools into clinical decision-making frameworks for neonatal sepsis. They proposed models for personalized EOS management, combining gestational age, perinatal stress exposure, immune biomarker profiles, and pathogen detection to guide individualized diagnostics and therapy, advocating for the development of bedside-compatible molecular diagnostic platforms, particularly to extend the benefits of accurate sepsis care into resource-limited settings [43,50].

## 4. Discussion

The present review synthesized molecular insights from 28 studies focused on early-onset neonatal sepsis (EOS), a condition whose pathophysiology remains only partially understood despite clinical urgency. Our findings confirm that numerous molecular pathways are implicated in EOS, including pathogen-induced inflammatory cascades, cytokine signaling, immune suppression, and emerging biomarker dynamics such as microRNAs and transcriptomic profiles.

A key challenge in EOS research lies in the heterogeneity of definitions and diagnostic criteria. While some studies included only culture-confirmed EOS cases, others relied on clinical criteria or surrogate markers such as elevated CRP, IL-6, or abnormal blood cultures without pathogen isolation. This diagnostic variability reflects real-world clinical practice, where microbiological confirmation is often lacking due to low sensitivity of blood cultures or early antibiotic administration. Accordingly, some studies—particularly observational cohorts—have emphasized the inflammatory phenotype of EOS rather than microbiological confirmation per se. Nonetheless, we acknowledge that this remains a point of ongoing debate in the field.

Moreover, the review highlights a wide variation in sample size, study design, and molecular targets. Several original studies employed small cohorts, limiting generalizability. Others were narrative or theoretical reviews, which provided conceptual depth but lacked empirical rigor. Thematic synthesis was made possible by reclassifying studies based on shared mechanistic domains rather than relying exclusively on study design. This approach allowed us to capture complementary perspectives, but also limited our ability to perform formal meta-analysis or assess heterogeneity in a quantitative manner.

This approach allowed us to capture complementary perspectives, but also limited our ability to perform formal meta-analysis or assess heterogeneity in a quantitative manner.

Although Dong et al. [32] and Vincent [41] do not focus exclusively on early-onset neonatal sepsis (EOS), they were included due to their relevance for understanding broader inflammatory and immune response mechanisms. Dong et al. [32] provide a conceptual framework for neonatal inflammation that intersects with EOS-related pathways, while Vincent [41] discusses systemic sepsis responses applicable to perinatal contexts. These studies were used for theoretical orientation only and did not contribute primary data to the synthesis.

This classification shows that most studies converged on pathogen recognition and systemic inflammatory responses.

### 4.1. Expanding the Understanding of EOS Pathogenesis

Across the reviewed studies, Toll-like receptors (TLRs) remain firmly established as key initiators of neonatal immune responses following pathogen exposure. However, the findings also highlight that the neonatal immune system is uniquely adapted for early life, though often inadequately prepared to control invasive infection. Preterm neonates in particular demonstrate a diminished capacity to produce effective responses to bacterial ligands such as lipopolysaccharide (LPS) and lipoteichoic acid. This decreased recognition can lead to delayed pathogen clearance, followed by exaggerated systemic inflammation once inflammatory thresholds are breached. These observations are consistent with the previous literature, that has documenting the increased vulnerability to sepsis among preterm populations [31,38].

### 4.2. The Role of Non-Infectious Inflammation

One critical insight is the significant contribution of sterile inflammation, initiated by hypoxia, oxidative stress, or mechanical injury, in driving sepsis-like clinical presentations. Studies highlight that EOS can occur even in the absence of microbiological confirmation, particularly among neonates exposed to antenatal stressors, challenging traditional diagnostic frameworks that prioritize pathogen detection and raising important considerations about the potential for overtreatment with empirical antibiotics. Moreover, several studies have described the simultaneous presence of systemic inflammation and features of immune suppression, a phenomenon referred to as “sepsis-induced immunoparalysis”. The coexistence of elevated cytokines with T-cell and monocyte exhaustion suggests that therapeutic strategies targeting immune modulation may be necessary alongside antimicrobial therapy [40,41].

### 4.3. Interactions Between Infectious and Sterile Triggers

The reviewed literature also supports an integrated model of EOS pathogenesis, where infectious and sterile triggers act synergistically against a backdrop of neonatal developmental immaturity. Rather than viewing infection and sterile injury as distinct pathways, the findings point toward significant interaction and amplification between these mechanisms. Emerging bioinformatic and transcriptomic analyses suggest that neonates with EOS may express distinct molecular subtypes, characterized by differential regulation of inflammatory, immune, and metabolic pathways. These molecular insights pave the way for stratified risk assessment models and may eventually guide individualized therapeutic approaches tailored to a neonate’s specific immunological profile.

### 4.4. Diagnostic Challenges and Clinical Implications

Despite advances in our understanding of EOS pathophysiology, diagnostic challenges persist. Standard biomarkers such as C-reactive protein (CRP) and procalcitonin (PCT) continue to lack specificity, often failing to distinguish between infectious and sterile inflammatory responses. While emerging biomarkers like presepsin and circulating miRNAs show promise, further validation and integration into clinical contexts are needed. The findings emphasize the importance of developing complex biomarker panels that can simultaneously capture signals of both infectious and sterile origin, improving diagnostic accuracy while minimizing unnecessary antibiotic exposure. Clinically, this highlights the need for a paradigm shift, from a binary infection/ no-infection approach toward a more nuanced interpretation of neonatal immune activation within the broader context of perinatal stress and developmental stage.

### 4.5. Strengths and Limitations

This review draws strength from its comprehensive inclusion of molecular studies, clinical research, and systematic and narrative reviews, offering a broad overview of EOS pathogenesis. Nevertheless, certain limitations should be acknowledged. Many original studies were constrained by small sample sizes, variability in outcome definitions, and differences in methodological approaches. Additionally, while narrative reviews enriched thematic understanding, they often lacked systematic rigor or critical appraisal frameworks. Despite these limitations, the consistency of findings across diverse study types strengthens the overall conclusions and highlights common relevant themes.

## 5. Conclusions

This systematic review emphasizes the advancing understanding of EOS as a complex, multifactorial syndrome shaped by the interaction between microbial invasion, host immune immaturity, and non-infectious inflammatory stimuli. Rather than being solely the result of infection, emerging molecular evidence supports a more integrated model where pathogen-associated molecular patterns (PAMPs) and damage-associated molecular patterns (DAMPs) converge to drive immune activation and systemic inflammation [62,63,64]. This challenges traditional diagnostic patterns and encourages the implementation of mechanical contexts that combine immune profiling, molecular diagnostics, and individualized perinatal context [4,65,66].

Despite variability in study size and methodological approaches, the included studies provided a sufficiently rigorous evidence base for thematic synthesis. Most original research articles and systematic reviews demonstrated a low risk of bias, while smaller observational and narrative studies introduced modest limitations, primarily related to reporting clarity and transparency, reinforcing the central role of immune activation, endothelial dysfunction, and sterile inflammatory processes in shaping EOS pathophysiology, particularly among preterm and vulnerable neonatal populations [62,63,64,67].

Future research efforts should prioritize the development and validation of multi-marker diagnostic panels able of differentiating infectious from non-infectious inflammation in EOS. Integrating transcriptomic, proteomic, and clinical data could simplify the identification of immunological endotypes, refining risk stratification and informing therapeutic thresholds. In parallel, targeted immunomodulatory strategies may offer alternatives to the usual reliance on broad-spectrum antibiotics. Achieving these advances will require both rigorous research methodologies and a change within neonatal care, embracing precision medicine approaches and moving beyond conventional, one-size-fits-all models of sepsis diagnosis and treatment [14,16,64,65,66].

## Data Availability

The data supporting the reported results can be obtained by contacting anca.vulcanescu@umfcv.ro and mirela.siminel@umfcv.ro.

## Supplementary Materials

The following supporting information can be downloaded at: https://www.mdpi.com/article/10.3390/jcm14155315/s1, Supplementary Materials S1: Extended search strategy; Supplementary Materials S2: Risk of bias assessment; Supplementary Materials S3: PRISMA_2020_checklist [68].
