# Supplementary material for: Molecular Mechanisms Underlying Inflammation in Early-Onset Neonatal Sepsis: A Systematic Review of Human Studies
Source: J Clin Med. 2025 Jul 28;14(15):5315. doi: 10.3390/jcm14155315 (PMC12347463; doi:10.3390/jcm14155315)
Supplement: Supplementary file 1 [file jcm-14-05315-s001.zip › Supplementary material S1 - extended search strategy.pdf]

Supplementary material 1. **Extended search strategy**

PubMed
A combination of MeSH terms and free-text keywords were used to capture studies on molecular mechanisms in early-onset neonatal sepsis. Boolean operators and filters were applied to narrow results:
("neonatal sepsis"[MeSH Terms] OR "neonatal sepsis"[tiab] OR "early-onset sepsis"[tiab] OR "early onset neonatal sepsis"[tiab] OR "EOS"[tiab]) AND ("molecular mechanisms"[tiab] OR "pathogenesis"[tiab] OR "immune response"[tiab] OR "cytokines"[tiab] OR "toll-like receptors"[tiab] OR "TLR"[tiab] OR "DAMPs"[tiab] OR "sterile inflammation"[tiab]) AND ("humans"[MeSH Terms]) AND ("infant, newborn"[MeSH Terms] OR neonate[tiab] OR newborn[tiab]) AND ("2015/01/01"[PDAT] : "2025/01/01"[PDAT]) AND (english[lang])

Filters Applied:

- Publication date: 2015–2025
- Language: English
- Species: Humans
- Age group: Newborns (0–28 days)
- Study types: Original research, reviews (manual inclusion criteria)

Initial results were reviewed by title and abstract. Studies focusing solely on animal models or lacking molecular focus were excluded. Manual de-duplication and full-text screening were completed prior to inclusion.

Scopus (Elsevier)
Scopus syntax and indexing was used with Boolean logic for broad retrieval. Keywords were searched in titles, abstracts, and keywords (TITLE-ABS-KEY):
TITLE-ABS-KEY("neonatal sepsis" OR "early-onset neonatal sepsis" OR "EOS") AND TITLE-ABS-KEY("molecular mechanisms" OR "immune response" OR "cytokine*" OR "toll-like receptor*" OR "TLR*" OR "DAMP*" OR "sterile inflammation") AND TITLE-ABS-KEY("neonate*" OR "newborn") AND (PUBYEAR > 2014 AND PUBYEAR < 2026) AND (LIMIT-TO(LANGUAGE, "English")) AND (DOCTYPE(ar) OR DOCTYPE(re))

Filters Applied:

- Year: 2015 to 2025
- Language: English
- Document type: Articles and reviews only
- Fields: Title, Abstract, Keywords

Results were exported and screened for relevance based on inclusion criteria. Studies without neonatal subjects or those not involving molecular analysis were excluded. Duplicates were removed prior to full-text assessment.

Web of Science (Core Collection)
The search was performed using the Topic (TS) field, covering titles, abstracts, author keywords, and Keywords Plus:
TS=("neonatal sepsis" OR "early-onset sepsis" OR "early-onset neonatal sepsis" OR "EOS") AND TS=("molecular mechanism*" OR "immune response" OR "cytokine*" OR "toll-like receptor*" OR "TLR*" OR "DAMP*" OR "sterile inflammation") AND TS=("neonate*" OR "newborn") AND LANGUAGE: (English) AND DOCUMENT TYPES: (Article OR Review) AND PUBLICATION YEARS: (2015–2025)

Filters Applied:

- Timespan: 2015 to 2025
- Language: English
- Document type: Article, Review
- Categories: Pediatrics, Immunology, Microbiology, Molecular Biology (used for manual triage)

Retrieved studies were exported and screened by two reviewers. Inclusion was based on molecular focus and relevance to EOS in human neonates. Articles that focused on late-onset sepsis or animal-only research were excluded.

Google Scholar

Google Scholar was used to supplement the peer-reviewed databases and ensure coverage of gray literature, recent publications not yet indexed elsewhere, and relevant biomedical theses and preprints.

Due to Google Scholar's limitations in using Boolean logic and controlled vocabulary, searches were performed using combinations of key terms and filters manually applied to refine results. The following search strings were used across several iterations: "early-onset neonatal sepsis" AND ("molecular mechanisms" OR "immune response" OR "cytokines" OR "toll-like receptors" OR "TLR" OR "DAMPs" OR "sterile inflammation") AND (neonate OR newborn)

Filters Applied:

- Custom date range: 2015–2025
- Language filter: English (manually selected where possible)
- Document types included: Peer-reviewed articles, conference papers, theses, preprints (selected based on relevance and quality)
- Manual exclusion: Animal-only studies, irrelevant disease models, duplicates

The first search results were screened for eligibility based on title and abstract. Full-text assessment was performed when abstracts were ambiguous or insufficient. Studies were included if they were original research or reviews with a clear focus on human neonates and molecular or immunologic mechanisms relevant to EOS.
