# Supplementary material for: Molecular Mechanisms Underlying Inflammation in Early-Onset Neonatal Sepsis: A Systematic Review of Human Studies
Source: J Clin Med. 2025 Jul 28;14(15):5315. doi: 10.3390/jcm14155315 (PMC12347463; doi:10.3390/jcm14155315)
Supplement: Supplementary file 1 [file jcm-14-05315-s001.zip › Supplementary material S2. Risk of bias assessment.pdf]

Supplementary material 2. **Risk of bias assessment**

Study	Study Design	Sample Size	Outcome Reporting	Funding Disclosure	Conflict of Interest	Overall Bias
**Marchant et al., 2015**	Observational	Moderate	Clear	Yes	No	Low
**Nakstad et al., 2016**	Experimental (in vitro)	Not applicable	Clear	Yes	No	Moderate
**Dias et al., 2021**	Narrative Review	Not applicable	Partial	No	Not reported	Moderate
**Shane et al., 2017**	Narrative Review	Not applicable	Clear	Yes	No	Low
**Dong & Speer, 2015**	Narrative Review	Not applicable	Partial	No	Not reported	Moderate
**Sweeney et al., 2017**	Narrative Review	Not applicable	Clear	Yes	No	Low
**Raymond et al., 2017**	Observational	Moderate	Clear	Yes	No	Low
**Khaertynov et al., 2017**	Observational	Small	Partial	Yes	No	Moderate
**Wynn & Wong, 2016**	Narrative Review	Not applicable	Clear	Yes	No	Low
**Ershad et al., 2019**	Narrative Review	Not applicable	Clear	No	Not reported	Moderate
**Speer, 2019**	Narrative Review	Not applicable	Clear	Yes	No	Low
**Hibbert et al., 2018**	Observational	Moderate	Clear	Yes	No	Low
**Moon et al., 2021**	Observational	Small	Partial	Yes	No	Moderate
**Vincent, 2023**	Narrative Review	Not applicable	Clear	No	Not reported	Low
**Wynn & Polin, 2018**	Narrative Review	Not applicable	Clear	Yes	No	Low
**Conti et al., 2020**	Narrative Review	Not applicable	Clear	Yes	No	Moderate
**Parra-Llorca et al., 2023**	Observational	Moderate	Clear	Yes	No	Low
**Tsantes et al., 2023**	Narrative Review	Not applicable	Clear	Yes	No	Low
**Gialamprinou et al., 2023**	Observational	Moderate	Clear	Yes	No	Moderate
**Hensler et al., 2022**	Observational	Moderate	Clear	Yes	No	Low
**Yan & Zhou, 2022**	Bioinformatics	N/A (database)	Partial	No	Not reported	Moderate
**Luo et al., 2023**	Bioinformatics	N/A (database)	Clear	Yes	No	Low
**Celik et al., 2022**	Narrative Review	Not applicable	Clear	Yes	No	Low
**Ruan et al., 2018**	Meta-analysis	Large	Clear	Yes	No	Moderate
**Jouza et al., 2022**	Observational	Small	Partial	Yes	No	Moderate
**Ng et al., 2015**	Narrative Review	Not applicable	Clear	No	Not reported	Low
**Chauhan et al., 2017**	Narrative Review	Not applicable	Partial	No	Not reported	Moderate
**Pietrasanta et al., 2019**	Narrative Review	Not applicable	Clear	No	No	Low

1. Study Design

Among the 28 included studies, 14 were original research articles, 5 were systematic or meta-reviews, and 9 were narrative reviews. The majority of original studies were observational, either prospective or retrospective cohorts. These were appropriate designs given the clinical and ethical constraints in studying EOS in human neonates. The inclusion of in vitro and bioinformatics studies was considered acceptable if human clinical relevance was explicitly addressed. Narrative reviews were heterogeneous in scope and methodology. While several (e.g., Shane et al., 2017; Speer, 2019) offered comprehensive and structured insight, others lacked defined inclusion criteria or methods for evidence selection.

2. Sample Size

Sample sizes varied significantly among the original studies. Studies such as Marchant et al. (2015) and Parra-Llorca et al. (2023) enrolled moderate cohorts, providing reasonable external validity. Others, like Moon et al. (2021) or Jouza et al. (2022), were limited by small, single-center populations, potentially introducing sampling bias. No power calculations were provided in any of the small-sample studies, and confidence intervals were inconsistently reported, which affects reliability and statistical interpretability.

3. Outcome Reporting

Most original studies provided clear descriptions of molecular or immunologic endpoints (e.g., cytokine levels, gene expression, receptor activation), with outcomes relevant to early-onset neonatal sepsis. However, partial reporting of statistical analyses or failure to stratify by gestational age or pathogen burden was observed in several moderate-risk studies (e.g., Khaertynov et al., 2017; Yan & Zhou, 2022). Reviews varied: systematic reviews adhered to standard outcome reporting frameworks (e.g., Ruan et al., 2018), whereas some narrative reviews offered broad overviews without consistent outcome comparison or synthesis.

4. Funding Disclosure

Funding transparency was noted in 22 of the 28 studies. Among the reviews, several did not include funding declarations, particularly older narrative overviews. This limits the ability to assess the potential influence of industry or institutional bias. All original studies clearly disclosed their funding sources.

5. Conflict of Interest Declarations

Conflict of interest (COI) was well-reported in 75% of the included studies. All original research articles included COI statements; however, 5 narrative reviews and 1 bioinformatics article lacked clear COI disclosure. Absence of COI reporting in reviews can introduce uncertainty, especially when summarizing data that may influence clinical perspectives.

6. Overall Bias Rating

- Low Risk (n = 15): Studies with clear methodology, transparent reporting, adequate sample size or scope, and no conflict of interest concerns.
- Moderate Risk (n = 13): Studies with unclear funding, small sample size, or partial reporting of methods or outcomes, especially among narrative reviews and computational studies.

Despite some heterogeneity in design and quality, the included studies provide collectively robust evidence for characterizing molecular pathways in EOS.
